# Supplementary material for: Endosomal LC3C-pathway selectively targets plasma membrane cargo for autophagic degradation
Source: Nat Commun. 2022 Jul 2;13:3812. doi: 10.1038/s41467-022-31465-3 (PMC9250516; doi:10.1038/s41467-022-31465-3)
Supplement: Supplementary file 1 — Supplementary Information [file 41467_2022_31465_MOESM1_ESM.pdf]

Endosomal LC3C-pathway selectively targets plasma membrane cargo for autophagic degradation

Paula P. Coelho, Geoffrey G. Hesketh, Annika Pedersen, Elena Kuzmin, Anne-Marie N. Fortier, Emily S. Bell, Colin D.H. Ratcliffe, Anne-Claude Gingras, Morag Park

SUPPLEMENTARY INFORMATION:

Supplementary Figure Legends and Figures 1-7.

Supplementary Tables 1-3.

Supplementary Figure 1

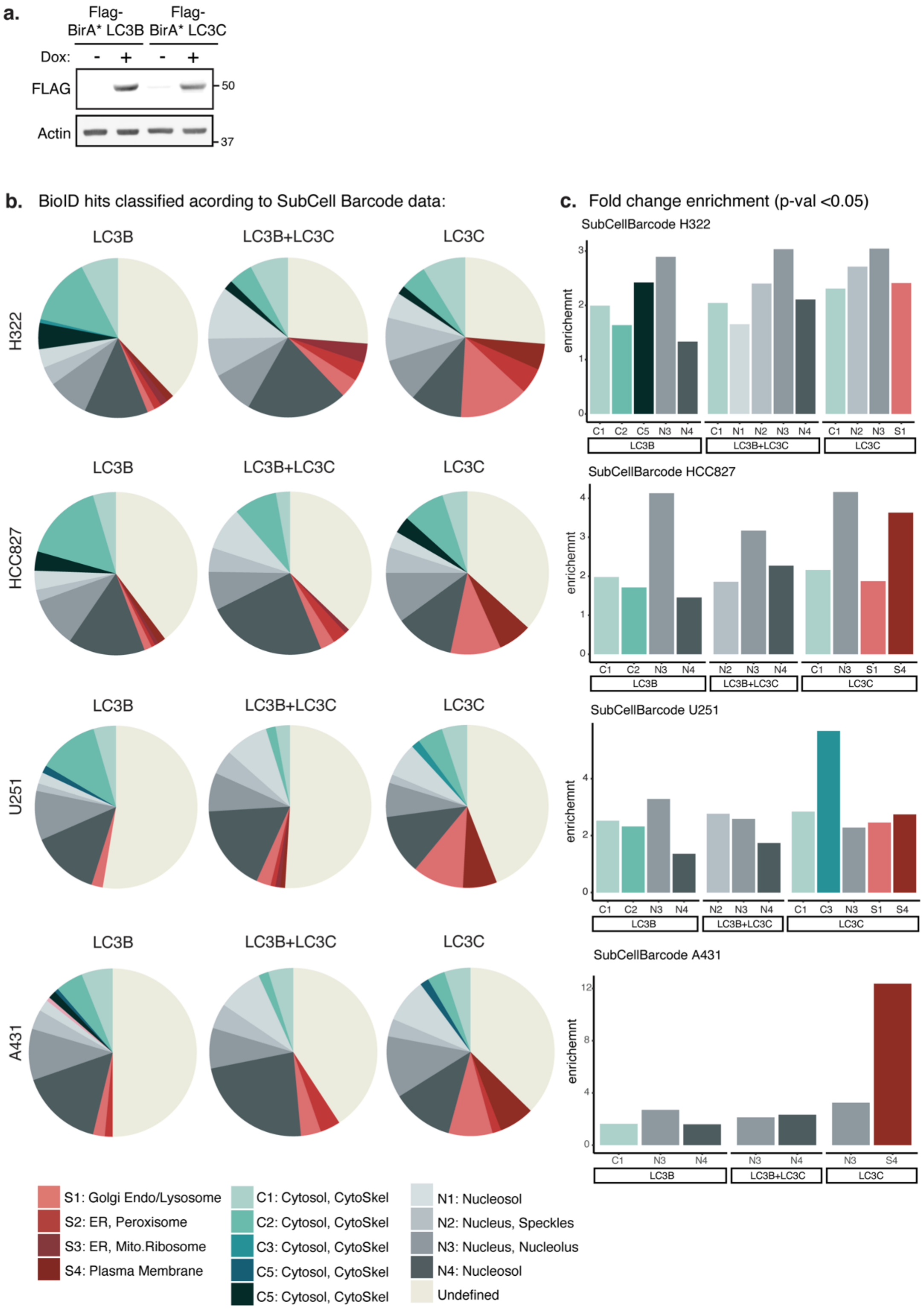

Supplementary Fig. 1 – LC3C-proximal interactome shows significant enrichment for proteins localized at the PM and endosomes. Related to Figure 1.

a. Western blot analysis of expression levels of BirA*-FLAG-LC3B and BirA*-FLAG-LC3C following 24h induction by addition of 1 μg/mL tetracycline. Representative image from two experimental replicates.

b. Classification of subcellular distribution of LC3 proximal interactors identified in the BioID screen according to SubCell Barcode database[32] from four different cancer cell lines (H322, HCC827, U251 and A431).

c. Fold change enrichment of different compartments among interactors of each bait used in the screen across the four different cancer cell lines from (B). Only compartments significantly enriched ($p < 0.05$) as assessed by a hypergeometric test are plotted.

Supplementary Figure 2

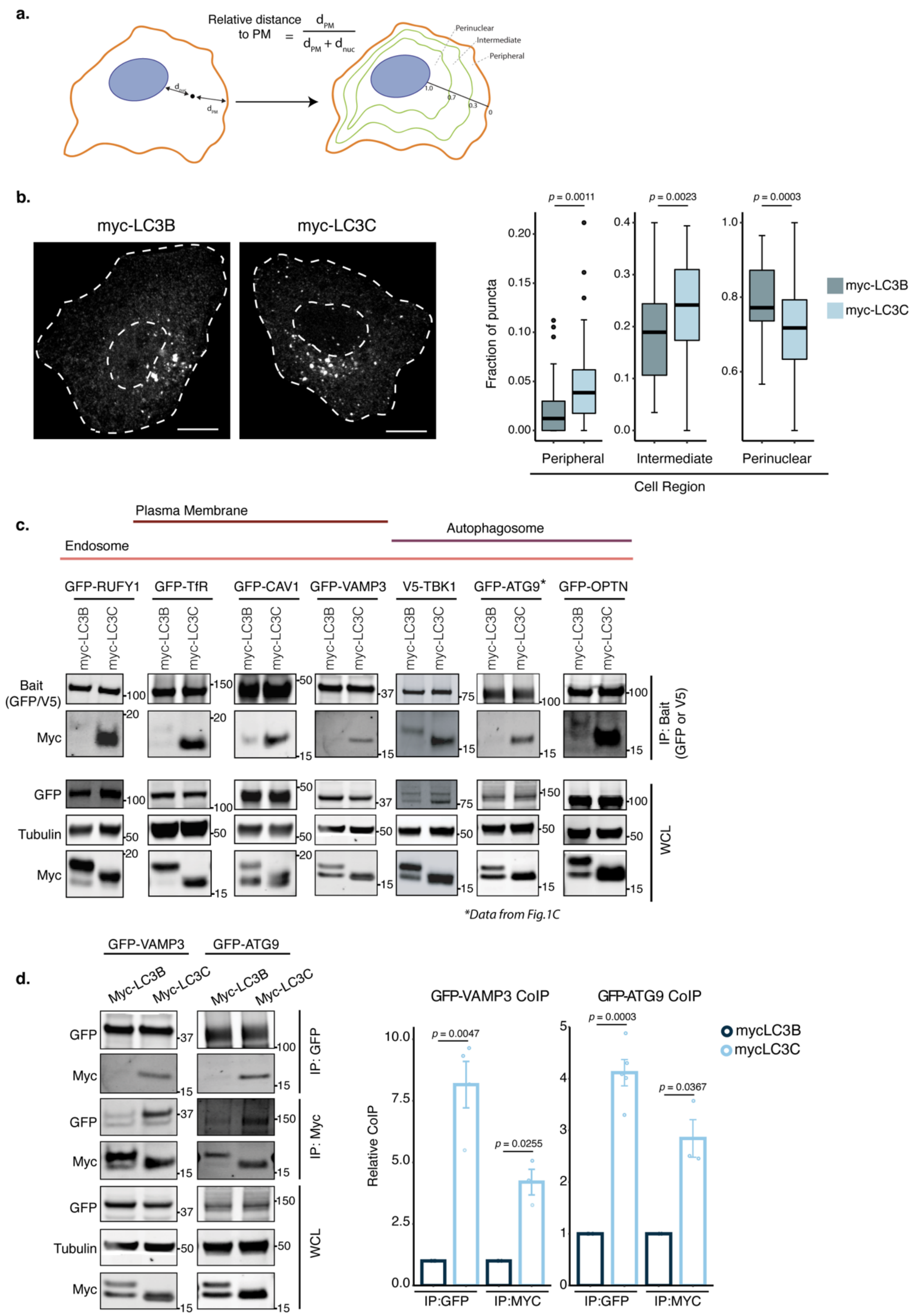

Supplementary Fig. 2 – LC3C selectively interacts with proteins which localize to endosomes, PM and autophagosomes. Related to Figure 2.

a. Schematic of quantification methodology used to calculate distribution profile. The distance to both the PM and nucleus is measured for each protein puncta detected by immunofluorescence. To take into account irregular cell shapes, the relative distance to the PM is then calculated for each puncta. Finally, the fraction of puncta across three subcellular regions: perinuclear, peripheral and intermediate is quantified to obtain a distribution profile.

b. Representative images of subcellular distribution of myc-LC3B and myc-LC3C transiently transfected into HeLa cells under basal conditions (Scale bar = 10 μm), with quantification on the left. Fraction of puncta identified across three subcellular cellular regions (peripheral, intermediate and perinuclear) are plotted. Box plots indicate median (middle line), 25th, 75th percentile (box) and 1.5 IQR (Interquartile range) of the nearer quartile (whiskers) and outliers (single points). 55-65 cells were quantified per condition across three experiments (unpaired two-sample t-test). Source data are provided as a Source Data file

c. Co-immunoprecipitation of predicted LC3C-specific proximal interactors annotated to localize at intersections of PM, endosomes and autophagosomes with myc-LC3C and myc-LC3B. Prior to lysis, cells were starved for 2h in HBSS. ($N=3$ for RUFY1, TfR, CAV1, TBK1, $N=4$ for OPTN, VAMP3 and $N=5$ for ATG9) See also Fig. 1e.

d. Forwards and reverse co-immunoprecipitation experiments of GFP-VAMP3 and GFP-ATG9 with myc-LC3C and myc-LC3B validating selective interaction of LC3C. Quantification of blots is shown on the right; display fold change increase in interaction of hits with myc-LC3C compared to myc-LC3B. ($N=4$ for GFP Co-IPs, $N=3$ for MYC Co-IPs, mean ± SEM, unpaired two-sample t-test). Source data are provided as a Source Data file

Supplementary Figure 3

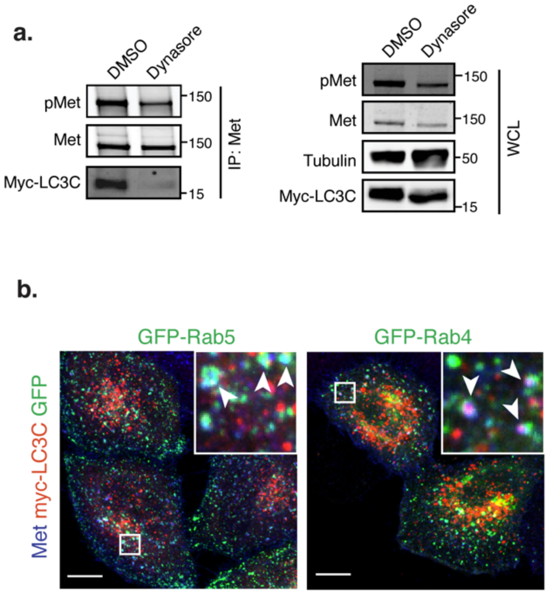

Supplementary Fig. 3 – LC3C interacts with Met following internalization, colocalizing in an endocytic compartment. Related to Figure 3.

a. HeLa cells transiently transfected with myc-LC3C were treated with 100µM Dynasore to block clathrin-mediated endocytosis, then starved and stimulated with HGF for 20min prior to lysis. Co-immunoprecipitation of Met and LC3C was then assessed, showing that dynasore treatment and blockage of Met-internalization abrogated Met-LC3C interactions. Representative image based on two experimental replicates.

b. HeLa cells were transfected with myc-LC3C and either early endosome marker, GFP-Rab5, or fast-recycling endosome marker, GFP-Rab4, starved and stimulated with HGF for 15min to trigger Met-internalization prior to fixation. Met-LC3C colocalization in endocytic structures is indicated by arrowheads for triple colocalized puncta. Scale bar = 20 µm. Representative images from one of three experimental replicates. Source data for quantification are provided as a Source Data file.

Supplementary Figure 4

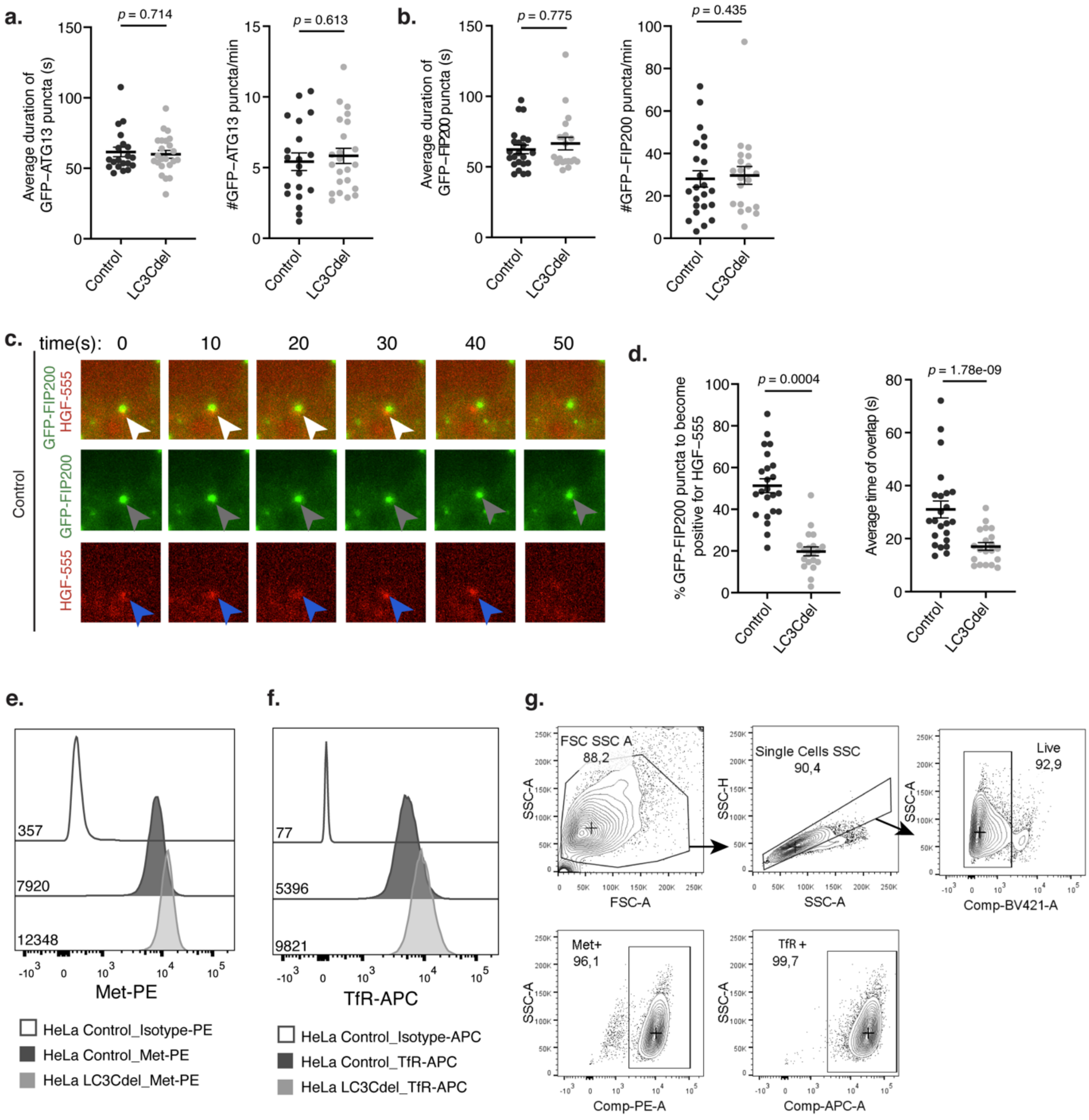

Supplementary Fig. 4 – LC3C deletion prevents Met-HGF complex from effectively reaching nascent phagophores. Related to Figure 4.

a. Quantification of GFP-ATG13 dynamics during live-cell imaging in control and cells with LC3C deletion (LC3Cdel). On the left, average duration GFP-puncta are shown and on the right the frequency of GFP puncta (defined as puncta/min) was calculated. 20 and 23 cells were quantified respectively for Control and LC3Cdel across two experimental replicates (mean ± SEM, unpaired two-sample t-test).

b. As in (b) quantification of GFP-FIP200 dynamics during live-cell imaging in control and LC3Cdel cells. 23 and 20 cells were quantified respectively for Control and LC3Cdel across two experimental replicates (mean ± SEM, unpaired two-sample t-test).

c. Representative images control cells expressing GFP-FIP200 starved for 1h in HBSS prior to stimulation with HGF-555 to visualize trafficking of the Met/HGF-555 complex (blue arrowhead) to GFP-FIP200-positive sites of autophagosome initiation (grey arrowhead). White arrowhead mark frames with colocalization.

d. Quantification of the experiment in (d). Graph on the left shows the proportion of GFP-FIP200 puncta that become positive for HGF-555. Graph on the right shows that the average duration of overlap between GFP-FIP200 and HGF-555 puncta. 23 and 20 cells were quantified respectively for Control and LC3Cdel across two experimental replicates (mean ± SEM, unpaired two-sample t-test).

e. Representative histogram of fluorescence intensity of cell surface Met antibody tagged with PE or isotype antibody tagged with PE in control and LC3Cdel cells. Mean Fluorescent Intensity (MFI) values are indicated on the left.

f. Representative histogram of fluorescence intensity of cell surface TfR antibody tagged with APC or isotype antibody tagged with APC in control and LC3Cdel cells. MFI values are indicated on the left.

g. Example of gating strategy used. Viable cells were first gated on a plot of FSC-A vs. SSC-A. Doublets were then excluded by subsequent gating on SSC-H vs. SSC-A plots. Live cells were identified using a cell viability dye. Met-PE of CD71-APC positive gating was determined based on isotype antibody control.

Source data for (a), (b) and (d) are provided as a Source Data file

Supplementary Figure 5

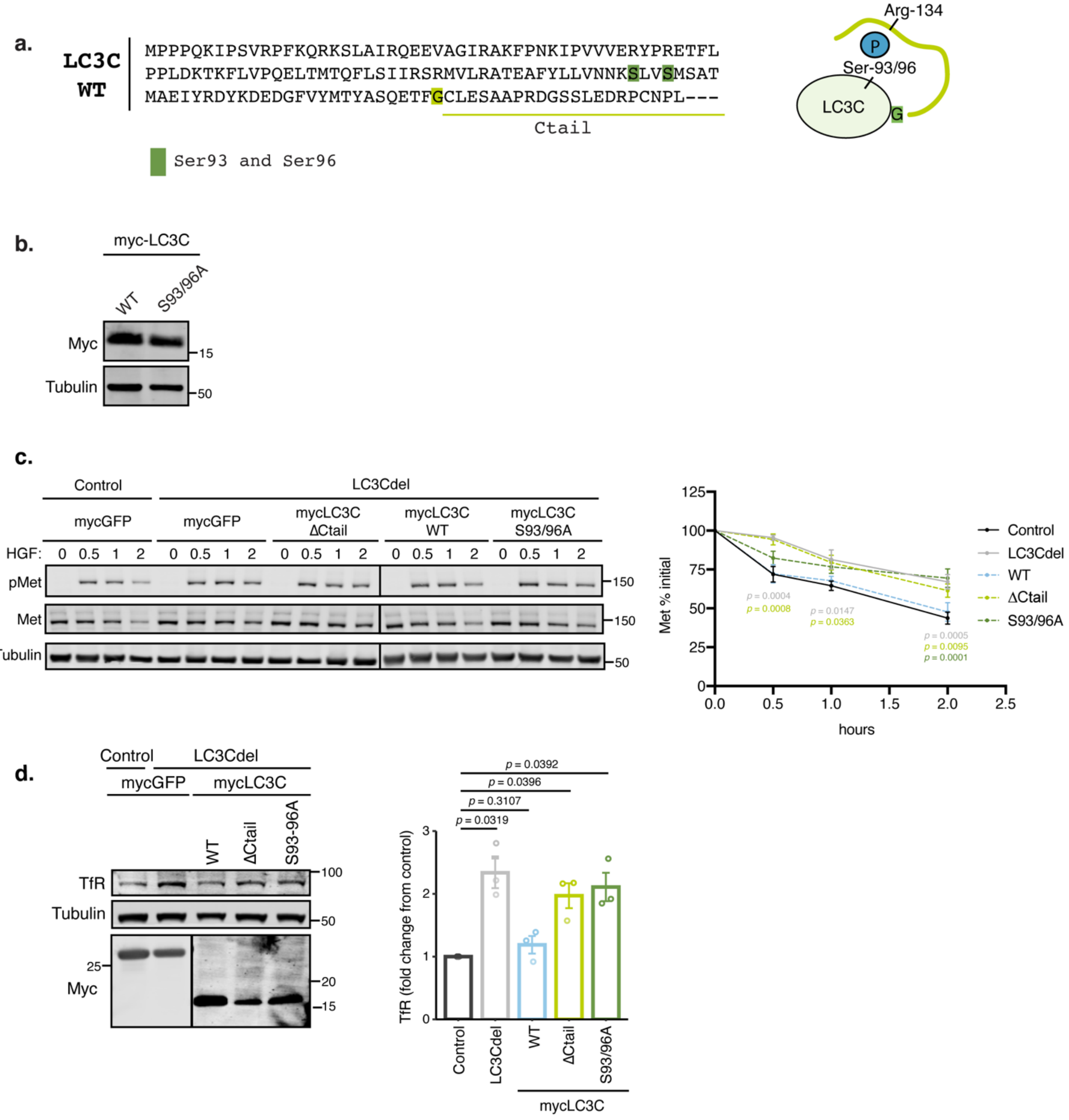

Supplementary Fig. 5 – the LC3C C-terminal tail and S93/96 phosphosites are necessary for LC3C dependent degradation of Met and TfR. Related to Figure 5.
a. On the left**,** schematic of LC3C depicting Ser93 and Ser96. On the right model of intramolecular interactions of phosphorylated Ser93/96 with the carboxy-tail of LC3C.
b. Western blot of HeLa cells transfected with either LC3C-WT or LC3C-S93/96A mutant, confirms similar expression of both constructs. See also FigS5d for additional repeats.
c. Hela control and LC3Cdel cells were transiently transfected with the indicated myc-tagged constructs, starved in HBSS with cycloheximide to inhibit protein translation and stimulated with HGF for the indicated time points. Cells were lysed and analyzed by western blot to assess Met levels ($N = 4$, mean ± SEM, two-way ANOVA, Dunnett's multiple comparison test, only $p < 0.05$ are indicated). Source data are provided as a Source Data file.
d. Hela control and LC3Cdel cells were transiently transfected with the indicated myc-tagged constructs, starved in HBSS with cycloheximide to inhibit translation, lysed and analyzed by western blot to assess TfR levels ($N = 3$, mean ± SEM, one sample t-test from a hypothetical value of 1.0). Source data are provided as a Source Data file.

Supplementary Figure 6

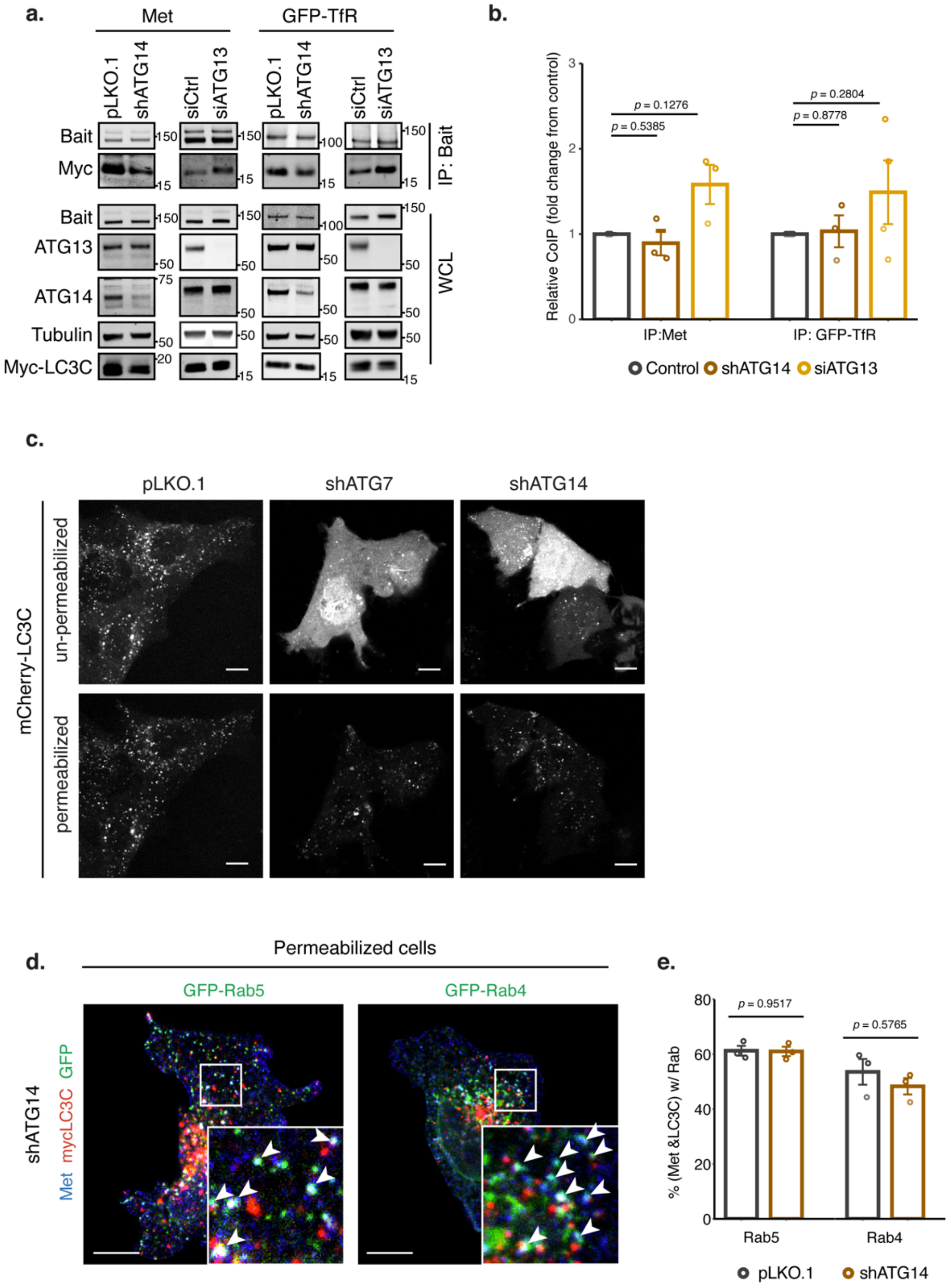

Supplementary Fig. 6 – LC3C interacts with cargo prior to autophagosome formation. Related to Figure 6.

a. Co-immunoprecipitation (CoIP) of endogenous Met or GFP-TfR with transiently transfected myc-LC3C following knockdown (KD) of ATG14 or ATG13. Cells were starved and, for the Met CoIPs, stimulated with HGF for 20min prior to lysis.

b. Quantification of western blot CoIP in (a). Graph displays fold change from control, either pLKO.1-empty vector control of siRNA-control as indicated (*N=3*, mean ± SEM, one sample t-test from a hypothetical value of 1.0). Source data are provided as a Source Data file

c. Representative image based on two experimental replicates of cells stably expressing shATG7, shATG14 or empty vector (pLKO.1) transiently transfected with mCherry-LC3C prior to and post cell permeabilization. Cells were starved for 2h in HBSS prior to imaging. Scale bar = 10 µm. See also Supplementary movie 1.

d. Representative images from one of three experimental replicates of cells with KD of ATG14 transfected with myc-LC3C and either early endosome marker, GFP-Rab5, or fast-recycling endosome marker, GFP-Rab4, starved for 2h in HBSS and stimulated with HGF for 15min to trigger Met-internalization prior to cell permeabilization and fixation. Arrowheads indicate triple colocalization of Met, myc-LC3C and GFP-Rab. Scale bar = 10 µm.

e. Quantification of immunofluorescence experiments in (d). Colocalization of Met and myc-LC3C with GFP-Rab5 or GFP-Rab4 was quantified in ATG14-KD compared to pLKO.1-empty vector controls treated and fixed in the same manner (*N=3,* values represent mean ± SEM, unpaired two-sample t-test). Source data are provided as a Source Data file

Supplementary Figure 7

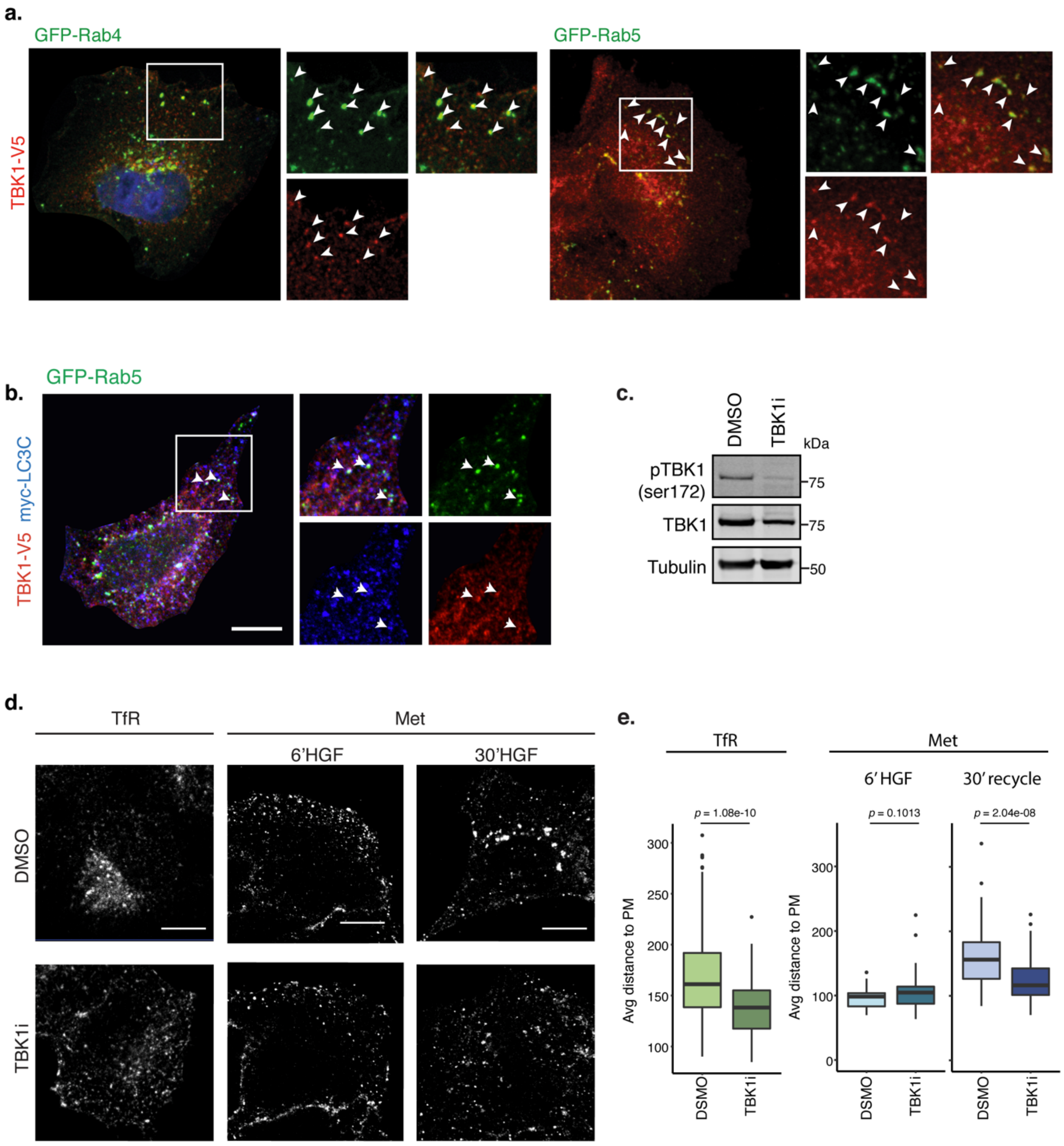

Supplementary Fig. 7 – Endocytic TBK1 colocalizes with LC3C and TBK1 inhibition impacts trafficking of LC3C-cargo, Met and TfR. Related to Figure 7.

a. Representative image based on three experimental replicates show colocalization V5-TBK1 and GFP-Rab4 or GFP-Rab5 in transiently transfected cells starved for 2h in HBSS and fixed prior to immunofluorescent staining for V5. Arrowheads indicate colocalization of V5-TBK1 and GFP-Rab in endosomal structures. Scale bar = 10 µm. Source data for quantification are provided as a Source Data file

b. Representative image based on three experimental replicates showing colocalization of V5-TBK1, GFP-Rab5 and myc-LC3C in transfected cells starved for 2h in HBSS and fixed prior to immunofluorescent staining for myc and V5. Arrowheads indicate colocalization of V5-TBK1, myc-LC3C and GFP-Rab. Scale bar = 10 µm. Source data for quantification are provided as a Source Data file.

c. Western-blot of cells treated with either DMSO or TBK1 small molecule inhibitor (TBK1i)-MRT67307 at 10uM. Decreased levels of phopho-TBK1(ser172) following treatment with TBK1i, confirm TBK1 inhibition. Representative image from two experimental replicates.

d. The effect of TBK1-inhibition on TfR and Met-trafficking was assessed. For TfR, cells were fixed following 2h treatment with TBK1-inhibition (10uM) or DMSO-control in HBSS and stained for endogenous TfR. For Met an immunofluorescence-based recycling assay was performed. Following 1.5h starvation in HBSS and treatment with TBK1i or DMSO, a short 6'HGF-pulse was used to induced Met internalization, after which cells were immediately fixed or submitted to a 30'chase period to allow for subsequent Met-trafficking and recycling. Scale bar = 10 µm. Source data are provided as a Source Data file

e. Quantification of immunofluorescence experiments from (d). Average puncta distance to the plasma membrane per cell was quantified for the indicated conditions. Box plots indicate median (middle line), 25th, 75th percentile (box) and 1.5 IQR (Interquartile range) of the nearer quartile (whiskers) and outliers (single points). For TfR, 115-140 cells were quantified per condition across four experiments; for Met following 30min recycling post-HGF-stimulation 90-110 cells were quantified per condition across five experiments and following 6min-HGF induced internalization 20-25 cells were quantified per condition across two experiments (unpaired two-sample t-test).

Supplementary Table 1 – Constructs

Construct name	Gene expressed	Source	Identifier/Ref
pcDNA3-Myc-LC3B	MAP1LC3B	Generated in the laboratories of Dr. Jayanta Debnath and Dr. Craig McCormick	NA
pcDNA3-Myc-LC3C	MAP1LC3C		
pEGFP-C1-ATG13	ATG13	Gift from Noboru Mizushima (Hosokawa et al., 2009)	Addgene; Plasmid #22875
pMXs-puro-RFP-ATG9A	ATG9	Gift from Noboru Mizushima (Koyama-Honda et al., 2013)	Addgene; Plasmid #60609
pLX_TRC317-NDP52-V5	CALCOCO2	Broad Human ORFeome v8.1 library - Sigma-Aldrich	TRCN0000478633
pEGFP-N1-OPTN	OPTN	Gift from Beatrice Yue (Park et al., 2006)	Addgene, Plasmid # 27052
pENTR223.1-FKBP15	FKBP15	Broad Human ORFeome v8.1 library - Sigma-Aldrich	Clone ID: BC166677
pLX_TRC317-BUB1-V5	BUB1	Broad Human ORFeome v8.1 library - Sigma-Aldrich	TRCN0000480863
pLX_TRC317-AAK1-V5	AAK1	Broad Human ORFeome v8.1 library - Sigma-Aldrich	TRCN0000489763
pLX_TRC317-COPG1-V5	COPG1	Broad Human ORFeome v8.1 library - Sigma-Aldrich	TRCN0000476630
pLX_TRC317-FAM134B	RETREG1	Broad Human ORFeome v8.1 library - Sigma-Aldrich	TRCN0000480130
pEGFP-C3-VAMP3	VAMP3	Gift from Thierry Galli (Galli et al.,1998)	Addgene; Plasmid #42310
pBa-EGFP-TfR	TFRC	Gift from Gary Banker & Marvin Bentley (Burack et al., 2000)	Addgene, Plasmid #45060
pLX_TRC317-TBK1-V5	TBK1	Broad Human ORFeome v8.1 library - Sigma-Aldrich	TRCN0000488336
pLX_TRC317-RUFY1-V5	RUFY1	Broad Human ORFeome v8.1 library - Sigma-Aldrich	TRCN0000469015

pMXs-IP-EGFP-hFIP200	RB1CC1	Gift from Noboru Mizushima (Hara et al., 2008)	Addgene, Plasmid # 38192
GFP-Rab4	RAB4A	GFP-Rab constructs were kindly provided by Dr. Stephen Ferguson and Dr. Robert Lodge	NA
GFP-Rab5	RAB5A		
pMD2.G packaging vectors	NA	Gift from Dr. Didier Trono	Addgene, plasmid #12259
psPAX2 packaging vectors	NA	Gift from Dr. Didier Trono	Addgene, plasmid #12260

Supplementary Table 2 – Oligonucleotids

shRNA vectors			
Gene targeted	Source	Identifier	Sequence
ATG9 #1	Mission®TRC genome-wide shRNA collections - Sigma-Aldrich	TRCN000 0148385	CCGGCTTTACGTCTATCCAGTCCTTCTCGAG AAGGACTGGATAGACGTAAAGTTTTTG
ATG9#2	Mission®TRC genome-wide shRNA collections - Sigma-Aldrich	TRCN000 0129286	CCGGCTTCCAGTACAAGGCAGTGTTCTCGA GAACACTGCCTTGTACTGGAAGTTTTTG
VAMP3#1	Mission®TRC genome-wide shRNA collections - Sigma-Aldrich	TRCN000 0029814	CCGGCGGGATTACTGTTCTGGTTATCTCGA GATAACCAGAACAGTAATCCCGTTTTT
VAMP3#2	Mission®TRC genome-wide shRNA collections - Sigma-Aldrich	TRCN000 0029815	CCGGGCAGCCAAGTTGAAGAGGAAACTCG AGTTTCCTCTTCAACTTGGCTGCTTTTT
ATG3	Mission®TRC genome-wide shRNA collections - Sigma-Aldrich	TRCN000 0148120	CCGGGATGTGACCATTGACCATATTCTCGA GAATATGGTCAATGGTCACATCTTTTTG
ATG5	Mission®TRC genome-wide shRNA collections - Sigma-Aldrich	TRCN000 0330392	CCGGCCTTTCATTCAGAAGCTGTTTCTCGAG AAACAGCTTCTGAATGAAAGGTTTTTG
ATG7	Mission®TRC genome-wide shRNA collections - Sigma-Aldrich	TRCN000 0007584	CCGGGCCTGCTGAGGAGCTCTCCATCTCGA GATGGAGAGCTCCTCAGCAGGCTTTTT

ATG14	Mission®TRC genome-wide shRNA collections - Sigma-Aldrich	TRCN0000142647	CCGGGTCTGGCAAATCTTCGACGATCTCGAGATCGTCGAAGATTTGCCAGACTTTTTG
ATG13	ON-TARGETplus ATG13 siRNA - SMARTpool from Dharmacon	L-020765-01-0005	

Primers used for cloning		
Construct generate	Primer	Sequence
pcDNA3-Myc-LC3C_S93-96A	LC3C_S93.96A_FW	GTCGCCATGAGCGCAACCATGGCA
	LC3C_S93.96A_RV	CAGGGCCTTGTTGTTCACCAGCAAGTAAAAG
pEGFP-C2-ATG9	HindIII-ATG9_FW	AAAAAAGCTTATGGCGCAGTTTGACACTGAATACCA
	ATG9-KpnI_RV	AAAACCGCGGCTATACCTTGTGCACCTGAGGGGGTAG
pEGFP-C2-FKBP15	EcoRI-FKBP15_FW	AAAAGAATTCATGTTCGGTGCGGGGGACGAG
	Kpn-FKBP15_RV	AAAAGGTACCTCCCAGCCAGTCAATGTCATC
pEGFP-C2-AAK1	EcoRI-AAK1_FW	AAAAGAATTCATGAAGAAGTTTTTCGACTCC
	KpnI-AAK1_RV	AAAAGGTACCGTCCAGGTCTATGAGCTGATC
pEGFP-N3-COPG1	XhoI-COPG1_FW	AAAACTCGAGATGTTGAAGAAATTCGACAAG
	BamHI-COPG1_RV	AAAAGGATCCTCCCACAGATGCCAAGATGAT
pEGFP-C2-RETREG1	EcoRI-FAM134B_FW	AAAAGAATTCATGCCTGAAGGTGAAGACTTT
	KpnI-FAM134B-RV	AAAAGGTACCATGGCCTCCCAGCAGATTTGA
pEGFP-N3-RUFY1	EcoRI-RUFY_FW	AAAAGAATTCATGATGGAGGAGCGTGCCAAC
	KpnI-RUFY_RV	AAAAGGTACCGGAGGCCGTGGAGGAGCAGCG
pmCherry-C2-LC3C	EcoRI-LC3C_FW	AAAAGAATTCATGCCGCCTCCACAGAAAAT
	BamHI-LC3C_RV	AAAAGGATCCCTAGAGAGGATTGCAGGGTC

Supplementary Table 3 – Antibodies

Antibody	Company/Origin	Catalog number, Reference	Dilution and use
mouse anti-c-Myc (9E10)	Takara Bio	Cat#(631206);	1:500 (western blot), 1:100 (immunofluorescence)
goat anti-Met (hHGFR)	R&D	Cat#(AF276), AB_355289	1:100 (immunofluorescence)
rabbit anti-Met (148)	In-house	N/A	1:1000 (western blot)
rabbit anti-Phospho-Met (Y1234/1235)	Cell Signaling	Cat#(3077), AB_2143884	1:1000 (western blot)
mouse anti-Tubulin	Sigma	Cat#(T5168), AB_477579	1:5000 (western blot)
rabbit anti-GFP	Thermo-Fisher Scientific	Cat#(A6455), AB_221570	1:1000 (western blot)
rabbit anti-GFP	Santa-Cruz	Cat#(sc-8334), AB_641123	1:1000 (western blot)
rabbit anti-ATG9	AbCam	Cat#(ab108338), AB_10863880	1:1000 (western blot), 1:75 (immunofluorescence)
rabbit anti-VAMP3	Cell Signaling	Cat#(13640S), AB_2798280	1:500 (western blot)
mouse anti-TfR	Cymbus Biotechnologies	Cat#(CBL137)	1:100 (immunofluorescence)
mouse anti-V5	AbCam	Cat#(ab27671), AB_471093	1:1000 (western blot)
rabbit anti-V5	Sigma	Cat#(V8137), AB_261889	1:1000 (western blot), 1:75 (immunofluorescence)
rabbit anti-TBK1	Cell Signaling	Cat#(38066), AB_2827657	1:500 (western blot)
rabbit anti-Phospho-TBK1(Ser172)	Cell Signaling	Cat#(5483),	1:500 (western blot)
rabbit anti-ATG3	Cell Signaling	Cat#(3415T), AB_2059244	1:1000 (western blot)
rabbit anti-ATG12	Cell Signaling	Cat#(2010S), AB_2059086	1:1000 (western blot)
rabbit anti-ATG7	Cell Signaling	Cat#(8558S), AB_10831194	1:1000 (western blot)
rabbit anti-ATG13	Cell Signaling	Cat#(13468T), AB_2797419	1:1000 (western blot)
rabbit anti-ATG14	Sigma	Cat#(A6358), AB_1852353	1:500 (western blot)
donkey anti-goat Alexa 647	Invitrogen (molecular probes)	Cat#(A21447), AB_141844	1:200 (immunofluorescence)

donkey anti-goat Alexa 555	Invitrogen (molecular probes)	Cat#(A21432), AB_2535853	1:500 (immunofluorescence)
donkey anti-rabbit Alexa 488	Invitrogen (molecular probes)	Cat#(A21206), AB_2535792	1:500 (immunofluorescence)
donkey anti-mouse Alexa 555	Invitrogen (molecular probes)	Cat#(A21422), AB_141822	1:500 (immunofluorescence)
donker anti-mouse Alexa 647	Invitrogen (molecular probes)	Cat#(A31571), AB_162542	1:200 (immunofluorescence)
goat anti-mouse IRDye 680RD antibody (odyssey)	Mandel Scientific	Cat#(LIC-926-68070)	1:10000 (western blot)
goat anti-rabbit IRDye 800CW antibody (odyssey)	Mandel Scientific	Cat#(LIC-926-32211)	1:10000 (western blot)
PE mouse anti-MET (clone 95106)	R&D systems	Cat#(FAB3582 P)	5 μl per million cells in 100 μl staining volume (flow)
APC mouse anti-CD71 (clone CY1G4)	Biolegend	Cat#(334108), AB_10915138	5 μl per million cells in 100 μl staining volume (flow)
