# Supplementary material for: Endosomal LC3C-pathway selectively targets plasma membrane cargo for autophagic degradation
Source: Nat Commun. 2022 Jul 2;13:3812. doi: 10.1038/s41467-022-31465-3 (PMC9250516; doi:10.1038/s41467-022-31465-3)
Supplement: Supplementary file 3 — Description of Additional Supplementary Files [file 41467_2022_31465_MOESM3_ESM.pdf]

Description of Additional Supplementary Files

File Name: Supplementary Data 1
Description: BioID data and analysis

File Name: Supplementary Movie 1
Description: **Cell permeabilization reveals presence of stable LC3C puncta in shATG7 cells.** HeLa cells stably expressing shATG7 were transiently transfected with mCherry-LC3C and starved for 2h in HBSS prior to imaging. Permeabilization buffer was added directly to cells during the imaging and unbound mCherry-LC3C can be seen to dissipate revealing the presence of vesicle bound mCherry-LC3C.
