# Supplementary material for: Endosomal LC3C-pathway selectively targets plasma membrane cargo for autophagic degradation
Source: Nat Commun. 2022 Jul 2;13:3812. doi: 10.1038/s41467-022-31465-3 (PMC9250516; doi:10.1038/s41467-022-31465-3)
Supplement: Supplementary file 6 — Reporting Summary [file 41467_2022_31465_MOESM6_ESM.pdf]

nature portfolio

Corresponding author(s): Morag Park

Last updated by author(s): Jun 14, 2022

Reporting Summary

Nature Portfolio wishes to improve the reproducibility of the work that we publish. This form provides structure for consistency and transparency in reporting. For further information on Nature Portfolio policies, see our Editorial Policies and the Editorial Policy Checklist.

Statistics

For all statistical analyses, confirm that the following items are present in the figure legend, table legend, main text, or Methods section.

n/a	Confirmed	
☐	☒	The exact sample size (n) for each experimental group/condition, given as a discrete number and unit of measurement
☐	☒	A statement on whether measurements were taken from distinct samples or whether the same sample was measured repeatedly
☐	☒	The statistical test(s) used AND whether they are one- or two-sided<br>*Only common tests should be described solely by name; describe more complex techniques in the Methods section.*
☒	☐	A description of all covariates tested
☐	☒	A description of any assumptions or corrections, such as tests of normality and adjustment for multiple comparisons
☐	☒	A full description of the statistical parameters including central tendency (e.g. means) or other basic estimates (e.g. regression coefficient) AND variation (e.g. standard deviation) or associated estimates of uncertainty (e.g. confidence intervals)
☐	☒	For null hypothesis testing, the test statistic (e.g. F, t, r) with confidence intervals, effect sizes, degrees of freedom and P value noted<br>*Give P values as exact values whenever suitable.*
☒	☐	For Bayesian analysis, information on the choice of priors and Markov chain Monte Carlo settings
☒	☐	For hierarchical and complex designs, identification of the appropriate level for tests and full reporting of outcomes
☒	☐	Estimates of effect sizes (e.g. Cohen's d, Pearson's r), indicating how they were calculated

Our web collection on statistics for biologists contains articles on many of the points above.

Software and code

Policy information about availability of computer code

Data collection	Mass spectrometry data was collected from TripleTOFTM 6600 instrument (AB SCIEX, Concord, Ontario, Canada). Confocal images were acquired on a Zeiss LSM800 laser scanning confocal microscope (Carl Zeiss) with a 63X objective. Lice cell images were captured with a TIRF-Spinning Disk Spectral Diskovery System (Spectral Applied Research, Richmond Hill, ON) based on a Leica DMI 6000 microscope stand (Quorum Technologies, Puslinch, ON) equipped with a Leica Plan-Apochromat 63x/1.47NA oil DIC objective, ImagEM X2 EM-CCD camera (Hamamatsu Photonics K.K., Hamamatsu City, Japan), and Chamlide CU-501 top-stage incubator system (Live Cell Instrument, Seoul, South Korea) using MetaMorph software (Molecular Devices) acquisition and assembly.
Data analysis	SAINTexpress (version 3.6.1 (45)) was used to score proximity interactions from MSPLIT-DIA data. Bait-vs-bait (bait versus bait) plots was generated using ProHits-viz70 (prohits-viz.org). Confocal images were analyzed using Zen software (Carl Zeiss; version 2.3) and MetaMorph software (Molecular Devices; version 7.7.7.0). Live Cell movie analysis was performed suing Cell Profiler software (McQuin et al., 2018; Carpenter et al., 2006, version 4.0.4). Flow cytometry data was analyzed with FlowJo (version X 10.0.7r2). Data were analyzed using R programming environment (v.3.6.2, r-project.org via R-Studio interface v.1.2.5033, rstudio.com) and visualized using the ggplot2 package (v.3.3.3, r-project.org).

For manuscripts utilizing custom algorithms or software that are central to the research but not yet described in published literature, software must be made available to editors and reviewers. We strongly encourage code deposition in a community repository (e.g. GitHub). See the Nature Portfolio guidelines for submitting code & software for further information.

Data

Policy information about availability of data

All manuscripts must include a data availability statement. This statement should provide the following information, where applicable:

- Accession codes, unique identifiers, or web links for publicly available datasets
- A description of any restrictions on data availability
- For clinical datasets or third party data, please ensure that the statement adheres to our policy

All MS files used in this study were deposited at MassIVE and have been assigned the following accession number: MSV000087983 (massive.ucsd.edu/ProteoSAFe/dataset.jsp?accession=MSV000087983). The ProteomeXchange accession is PXD027926. Source data for other experiments are provided with this paper.
Subcellular localization analysis was performed using data from the SubCellBarcode database (subcellbarcode.org). To increase coverage of sucellular location classification, BioID-hits were further annotated using Gene Ontology (GO)-term-name and GO-domain terms downloaded from BioMart (Esembl Gene 88; useast.ensembl.org/info/data/biomart/index.html), supplemented with information from GeneCards (genecards.org) and COMPARTMENTS database (compartments.jensenlab.org).

Field-specific reporting

Please select the one below that is the best fit for your research. If you are not sure, read the appropriate sections before making your selection.

[x] Life sciences [ ] Behavioural & social sciences [ ] Ecological, evolutionary & environmental sciences

For a reference copy of the document with all sections, see nature.com/documents/nr-reporting-summary-flat.pdf

Life sciences study design

All studies must disclose on these points even when the disclosure is negative.

Sample size	Sample size were based on previous experience with similar experimental systems (Parachoniak et al., Dev Cell 2011; Ratcliffe et al JCB 2019; Bell et all Cell Reports 2019), resulting in statistically meaningful comparisons.
Data exclusions	No data were excluded from analysis
Replication	All experimental data shown in the manuscript is the result of multiple independent experiments, as detailed in the figure legends and methods
Randomization	Randomization was not relevant for western blot analysis for controlled samples. For immunofluorescence experiments clusters of cells were chosen randomly for imaging using unbiased DAPI channel and all images were quantified (a minimum of 40 cells were quantified per condition across at least three experimental replicates). For live-cell imaging multiple transfected cells were randomly chosen for imaging.
Blinding	Blinding was not relevant for western blot analysis for controlled samples. For immunofluorescence experiments blinding was not relevant as cluster of cells were chosen for randomly imaging using unbiased DAPI channel, and all images were quantified.

Reporting for specific materials, systems and methods

We require information from authors about some types of materials, experimental systems and methods used in many studies. Here, indicate whether each material, system or method listed is relevant to your study. If you are not sure if a list item applies to your research, read the appropriate section before selecting a response.

Materials & experimental systems

n/a	Involved in the study	
[ ]	[x]	Antibodies
[ ]	[x]	Eukaryotic cell lines
[x]	[ ]	Palaeontology and archaeology
[x]	[ ]	Animals and other organisms
[x]	[ ]	Human research participants
[x]	[ ]	Clinical data
[x]	[ ]	Dual use research of concern

Methods

n/a	Involved in the study	
[x]	[ ]	ChIP-seq
[ ]	[x]	Flow cytometry
[x]	[ ]	MRI-based neuroimaging

Antibodies

Antibodies used	mouse anti-c-Myc (Takara Bio, #631206; WB-1:500, IF-1:100), goat anti-Met (R&D,#AF276; AB_355289; IF-1:100), rabbit anti-Met-148 (In-house, WB-1:1000), rabbit anti-Phospho-Met Y1234/1235(Cell Signaling, #3077, AB_2143884 (WB-1:1000), mouse anti-Tubulin (Sigma, #T5168, AB_477579, WB-1:5000) rabbit anti-GFP (Thermo-Fisher Scientific, #A6455, AB_221570, WB-1:1000), rabbit

anti-GFP (Santa-Cruz, #sc-8334, AB_641123, WB-1:1000), rabbit anti-ATG9 (AbCam, #ab108338, AB_10863880,WB-1:1000, IF-1:75), rabbit anti-VAMP3 (Cell Signaling, #13640S, AB_2798280, WB-1:500), mouse anti-TFRC (Cymbus Biotechnologies, #CBL137, IF-1:100), mouse anti-V5 (AbCam, #ab27671, AB_471093, WB-1:1000), rabbit anti-V5 (Sigma, #V8137, AB_261889, WB-1:1000, IF-1:75), rabbit anti-TBK1 (Cell Signaling, #38066, AB_2827657, WB-1:500), rabbit anti-Phospho-TBK1-Ser172 (Cell Signaling, #5483, WB-1:500), rabbit anti-ATG3 (Cell Signaling, #3415T, AB_2059244, WB-1:1000), rabbit anti-ATG7 (Cell Signaling, #8558S, AB_10831194, WB-1:1000), rabbit anti-ATG12 (Cell Signaling, #2010S, AB_2059086, WB-1:1000), rabbit anti-ATG13 (Cell Signaling, #13468T, AB_2797419, WB-1:1000), rabbit anti-ATG14 (Sigma, #A6358, AB_1852353, WB-1:500), donkey anti-goat Alexa 647 (Invitrogen/ molecular probes, #A21447, AB_141844, IF-1:200), donkey anti-goat Alexa 555 (Invitrogen/molecular probes, #A21432, AB_2535853, IF-1:200), donkey anti-rabbit Alexa 488 (Invitrogen/molecular probes, #A21206, AB_2535792, IF-1:500), donkey anti-mouse Alexa 555 (Invitrogen/molecular probes, #A21422, AB_141822, IF-1:500),donker anti-mouse Alexa 647 (Invitrogen/molecular probes, Cat#A31571, AB_162542, IF-1:200) ,goat anti-mouse IRDye 680RD antibody-odyssey (Mandel Scientific, #LIC-926-68070, WB-1:10000), goat anti-rabbit IRDye 800CW antibody-odyssey (Mandel Scientific, #LIC-926-32211, WB-1:10000), PE mouse anti-MET - clone 95106 (R&D systems, #FAB3582P, Flow-5 µl per million cells in 100 µl staining volume), APC mouse anti-CD71-clone CY1G4 (Biolegend, #334108, AB_10915138, Flow-5 µl per million cells in 100 µl staining volume), PE mouse IgG1 κ isotype control-clone MOPC-21 (Biolegend, #400114, Flow), APC mouse IgG2a κ isotype control-clone MOPC-173 (Biolegend, #400219, Flow). WB: western-blot, IF: immunofluorescence. Flow: flow-cytometry

Validation

1. The mouse anti-c-Myc (Takara Bio, #631206), from the Takara Bio website: Monoclonal antibody (Clone 9E10) raised against a synthetic peptide corresponding to residues 408-439 of the human p62-c-Myc protein. The antibody recognizes an epitope located within residues 410–419. This antibody is suitable for immunoprecipitation of c-myc tagged fusion proteins from cell lysates, ELISA, immunocytochemistry of microinjected or transfected cells, and localization of c-myc tagged proteins. We further validated antibody specificity by overexpression approaches. 2. The goat anti-Met (R&D#AF276), from the R&D systems website: Suitable for IF, IHC, WB. F. The antibody was knockout validated HGF R/c-MET is specifically detected in HeLa human cervical epithelial carcinoma parental cell line but is not detectable in HGF R/c-MET knockout HeLa cell line. 3. The rabbit anti-Met-148 (In-house): Anti-Met antibody (148) was generated by immunizing rabbits with the carboxy-terminal 16 amino acids (aa) of the human Met sequence, and previously described (Rodrigues GA, et al., Mol & Cell Biol 1991), and validated via overexpression approaches and using competing peptides to confirm signal specificity. 4. The rabbit anti-Phospho-Met Y1234/1235(Cell Signaling, #3077) from Cell Signaling website: Suitable for: WB, IP, IHC, IF, F. Phospho-Met Rabbit mAb detects endogenous levels of Met only when phosphorylated at Tyr1234/1235. Antibody validated via western blot analysis of cell extracts from HeLa cells, untreated or stimulated with HGF. 5. The mouse anti-Tubulin (Sigma, #T5168) from Sigma website: Recognizes an epitope located at the C-terminal end of the α-tubulin isoform in a variety of organisms. Monoclonal Anti-α-Tubulin antibody produced in mouse has been used in immunofluorescence Analysis, In western blotting/ Immunoblotting and For immunolabelling cells in electron microscopy. 6. The rabbit anti-GFP (Thermo-Fisher Scientific, #A6455) from Thermo-Fisher website: Antibody specificity was demonstrated by detection of different targets fused to GFP tag in transiently transfected lysates tested. We further validated antibody specificity by overexpression approaches. 7. The rabbit anti-GFP (Santa-Cruz, #sc-8334), from Santa-cruz website: epitope corresponding to amino acids 1-238 representing full length GFP. We further validated antibody specificity by overexpression approaches. 8. The rabbit anti-ATG9 (AbCam, #ab108338), from Abcam website: Suitable for: F, WB, IP, IHC, IF. Unpurified ab108338 was shown to specifically react with ATG9A when ATG9A knockout samples were used. We further validated antibody specificity by overexpression and knock-down approaches 9. The rabbit anti-VAMP3 (Cell Signaling, #13640S) from Cell Signaling website: Suitable for: WB and IP. VAMP3 recognizes endogenous levels of total VAMP3 protein. This antibody does not cross-react with VAMP1 or VAMP2 proteins. We further validated antibody specificity by overexpression and knock-down approaches. 10. The mouse anti-TFRC (Cymbus Biotechnologies, #CBL137): we validated antibody specificity by overexpression approaches. 11. The mouse anti-V5 (AbCam, #ab27671), from Abcam website: Suitable for: F, IHC, ELISA, IP, WB, RIA. ab27671 has been used to detect recombinant proteins, some of which include transmembrane and secreted proteins, which have been tagged with the V5 epitope. We further validated antibody specificity by overexpression approaches. 12. The rabbit anti-V5 (Sigma, #V8137), from Sigma website: Suitable for IHC, IP, WB. Recognizes V5 Tag (GKPIPNPLLGLDST) fusion proteins. We further validated antibody specificity by overexpression approaches. 13. The rabbit anti-TBK1 (Cell Signaling, #38066), from Cell Signaling website: Suitable for WB, IP, IF, F. Monoclonal antibody was validated for western blot analysis with extracts from HCT 116 cells, either wild-type (+/+) or TBK1/NAK knockout (-/-). 14. The rabbit anti-Phospho-TBK1-Ser172 (Cell Signaling, #5483), from Cell Signaling website: Suitable for WB, IP, IF, F. Monoclonal antibody was validated for WB with analysis of extracts from THP-1 cells differentiated with TPA #4174 (80 nM, overnight) followed by treatment with LPS (1 µg/ml), up to 24h, using Phospho-TBK1/NAK (Ser172).15. The rabbit anti-ATG3 (Cell Signaling, #3415T), from Cell Signaling website: Suitable for WB. Atg3 Antibody detects endogenous levels of total Atg3 protein. Antibody validated via western blot analysis of extracts from various cell lines. We further validated antibody specificity by knock-down approaches. 16. The rabbit anti-ATG7 (Cell Signaling, #8558S), from Cell Signaling website: Suitable for WB, IP. Antibody validated via western blot analysis of extracts from HeLa cells, transfected with 100 nM SignalSilence® Control siRNA (Unconjugated) #6568 (-) or SignalSilence® Atg7 siRNA. We further validated antibody specificity by knock-down approaches. 17. The rabbit anti-ATG12 (Cell Signaling, #2010S), from Cell Signaling website: Suitable for WB, IF. Atg12 Antibody (Human Specific) detects endogenous levels of total free and Atg5 bound Atg12 protein. Antibody validated via western blot analysis of extracts from various cell lines. We further validated antibody specificity by knock-down approaches 18. The rabbit anti-ATG13 (Cell Signaling, #13468T), from Cell Signaling website: Suitable for WB, IP, IF. Atg13 (E1Y9V) Rabbit mAb recognizes endogenous levels of total Atg13 protein. Antibody validated via western blot analysis of extracts from 293T cells, mock transfected (-) or transfected with a construct expressing Myc/DDK-tagged full-length human Atg13 (hAtg13-Myc/DDK; +). We further validated antibody specificity by knock-down approaches 19. The rabbit anti-ATG14 (Sigma, #A6358), from Sigma website: Anti-Atg14 recognizes human and mouse Atg14. The antibody may be used in several immunochemical techniques including immunoblotting. Detection of the Atg14 band by immunoblotting is specifically inhibited by the immunizing peptide. We further validated antibody specificity by knock-down approaches 20. The PE mouse anti-MET - clone 95106 (R&D systems, #FAB3582P), from R&D systems website: Suitable for F. Validated in MDA-MB-231 human breast cancer cell line was stained with Mouse Anti-Human HGF R/c-MET PE-conjugated Monoclonal Antibody (Catalog # FAB3582P, filled histogram) or isotype control antibody. We further validated specificity using isotype control antibody in our experiments. 21. The APC mouse anti-CD71-clone CY1G4 (Biolegend, #334108), from BioLegend website: The antibody was purified by affinity chromatography and conjugated with APC under optimal conditions. Each lot of this antibody is quality control tested by immunofluorescent staining with flow cytometric analysis. We further validated specificity using isotype control antibody in our experiments. WB: western-blot, IF: immunofluorescence, IP: immunoprecipitation, IHC: immunohistochemistry F: flow-cytometry

Eukaryotic cell lines

Policy information about cell lines

Cell line source(s)	HeLa cells were obtained from ATCC, HEK-293T cells were obtained from Frank Graham (McMaster University)
Authentication	The cells lines used were not authenticated
Mycoplasma contamination	The cells used tested negative for mycoplasm contamination
Commonly misidentified lines (See ICLAC register)	No commonly misidentified cell lines were used in this study.

Flow Cytometry

Plots

Confirm that:

- [x] The axis labels state the marker and fluorochrome used (e.g. CD4-FITC).
- [x] The axis scales are clearly visible. Include numbers along axes only for bottom left plot of group (a 'group' is an analysis of identical markers).
- [x] All plots are contour plots with outliers or pseudocolor plots.
- [x] A numerical value for number of cells or percentage (with statistics) is provided.

Methodology

Sample preparation	For staining the following fluorescent-labeled antibodies were used: PE anti-MET (clone 95106; #FAB3582P R&D systems), APC anti-CD71 (clone CY1G4; #334108 Biolegend), PE mouse IgG1 κ isotype control (clone MOPC-21; #400114 Biolegend) and APC mouse IgG2a κ isotype control (clone MOPC-173; #400219 Biolegend). A fixable live/dead dye was used to distinguish viable cells (#423113 Biolegend). Cell surface staining was performed in FACS buffer (PBS supplemented with 0.5% BSA and 2mM EDTA).
Instrument	Stained cells were acquired on a LSR Fortessa flow cytometer (BD Biosciences)
Software	Data were analyzed with FlowJo LLC software (BD Biosciences, version X 10.0.7r2).
Cell population abundance	All flow cytometry analyses were performed on samples containing a minimum of 10,000 cells as identified by the gating strategy outlined below.
Gating strategy	Viable cells were first gated on a plot of FSC-A vs. SSC-A. Doublets were then excluded by subsequent gating on SSC-H vs. SSC-A plots. Live cells were identified using a cell viability dye. Met-PE of CD71-APC positive gating was determined based on isotype antibody control.

- [x] Tick this box to confirm that a figure exemplifying the gating strategy is provided in the Supplementary Information.
